# Two Novel SNPs in *ATXN3* 3’ UTR May Decrease Age at Onset of SCA3/MJD in Chinese Patients

**DOI:** 10.1371/journal.pone.0117488

**Published:** 2015-02-17

**Authors:** Zhe Long, Zhao Chen, Chunrong Wang, Fengzhen Huang, Huirong Peng, Xuan Hou, Dongxue Ding, Wei Ye, Junling Wang, Qian Pan, Jiada Li, Kun Xia, Beisha Tang, Tetsuo Ashizawa, Hong Jiang

**Affiliations:** 1 Department of Neurology, Xiangya Hospital, Central South University, Changsha, Hunan 410008, P. R. China; 2 Key Laboratory of Hunan Province in Neurodegenerative Disorders, Central South University, Changsha, Hunan 410008, P. R. China; 3 State Key Lab of Medical Genetics, Central South University, Changsha, Hunan 410078, P. R. China; 4 Department of Neurology, University of Florida, Gainesville, Florida, United States of America; 5 Department of Neurology, Xiangtan Central Hospital, Xiangtan, Hunan 411100, P. R. China; 6 Department of Neurology & Institute of Translational Medicine at University of South China, the First People’s Hospital of Chenzhou, Chenzhou, Hunan 423000, P. R. China; Institute of Health Science, CHINA

## Abstract

Spinocerebellar ataxia type 3 (SCA3), or Machado—Joseph disease (MJD), is an autosomal dominantly-inherited disease that produces progressive problems with movement. It is caused by the expansion of an area of CAG repeats in a coding region of *ATXN3*. The number of repeats is inversely associated with age at disease onset (AO) and is significantly associated with disease severity; however, the degree of CAG expansion only explains 50 to 70% of variance in AO. We tested two SNPs, rs709930 and rs910369, in the 3’ UTR of *ATXN3* gene for association with SCA3/MJD risk and with SCA3/MJD AO in an independent cohort of 170 patients with SCA3/MJD and 200 healthy controls from mainland China. rs709930 genotype frequencies were statistically significantly different between patients and controls (*p* = 0.001, *α* = 0.05). SCA3/MJD patients carrying the rs709930 A allele and rs910369 T allele experienced an earlier onset, with a decrease in AO of approximately 2 to 4 years. The two novel SNPs found in this study might be genetic modifiers for AO in SCA3/MJD.

## Introduction

Spinocerebellar ataxia type 3 (SCA3), or Machado—Joseph disease (MJD), is an autosomal dominantly-inherited polyglutamine (polyQ) disease. It presents heterogeneous clinical features, such as progressive cerebellar ataxia, external ophthalmoplegia, dysarthria, dysphagia, pyramidal signs, dystonia, rigidity, and distal muscle atrophy [1]. In general, SCA3/MJD is the most common autosomal dominant spinocerebellar ataxia in China, accounting for 62.09% of cases [2]. It is caused by the expansion of CAG repeats within the *ATXN3* coding region: normal individuals have 12 to 40 repeats, while patients have 51 to 86. The age at onset (AO) of SCA3/MJD ranges from 4 years to 75 years; mean AO is around 40 years [3, 4]. In patients, the number of CAG repeats is inversely associated with AO of SCA3/MJD and significantly affect disease severity [5, 6]. However, only 50 to 70% of variance in AO can be ascribed to the degree of CAG expansion, suggesting other factors may modulate AO [7, 8].

In a previous study, we used the miRCURY LNA Array (Exiqon, Denmark) to assay miRNA expression levels in serums from SCA3/MJD patients, finding that the miR-25 expression levels were significantly different than those of healthy controls [9]. Subsequently, *in vitro*, we confirmed that miR-25 directly targets sequences at position 259–266 of the *ATXN3* 3’UTR and down-regulates the aggregation of polyQ-expanded ataxin-3 protein (officially accepted by FEBS Lett, 2014). Accordingly, we used 170 SCA3/MJD patients and 200 healthy controls to look for mirSNPs in the miR-25 gene or in miR-25 target sequences in the 3’ UTR of the *ATXN3* gene related to the results above. We did not find any miR-25 related mirSNPs. However, we did find two SNPs, rs910369 and rs709930, located 107 base pairs upstream and 108 base pairs downstream from the miR-25 target sequences in the 3’ UTR of the *ATXN3* gene respectively: the two SNPs were in complete linkage disequilibrium (D’ = 1.000), as identified by SHEsis (http://analysis.bio-x.cn/myAnalysis.php). We tested the two SNPs for a statistical association with SCA3/MJD, specifically with AO of SCA3/MJD patients.

## Methods

### Subjects and materials

In our study, we recruited 170 unrelated Chinese SCA3/MJD patients (97 males and 73 females) from the outpatient neurology clinic of Xiangya Hospital, Central South University, in Hunan, China. Mean age of patients was 42.91±11.18 years old (range from 18 to 75 years old). This case-control study included 200 healthy controls matched for age, gender, ethnicity and area of residence. Written informed consent was obtained from all individuals. The study was approved by the Expert Committee of Xiangya Hospital of Central South University (equivalent to an Institutional Review Board).

Clinical data were collected, including AO, clinical presentations, and duration of SCA3/MJD (Table 1). Genomic DNA was extracted from all samples according to a standard protocol [10]. The (CAG)n tract size was determined for all SCA3/MJD patients by T-vector cloning and direct DNA sequencing. The miR-25 target site in the 3’ UTR of *ATXN3* were amplified using a pair of primers designed using Primer3 (http://bioinfo.ut.ee/primer3-0.4.0/): 5’-GGCAGCTGTGACCATGTC-3’ (forward) and 5’-AGCATCTGGGAAAGCACATG-3’ (reverse). The amplification reactions contained 1 μL genomic DNA (50 ng/μL), 0.1 μL LA Taq DNA Polymerase (TaKaRa, Japan), 0.4 μL dNTPs, 0.2 μL of each primer, 3.1 μL sterile water and 5 μL 2×GC Buffer I (TaKaRa, Japan), for a total of 10 μL. Genomic DNA was amplified by polymerase chain reaction (PCR) performed in Mastercyclers (Eppendorf AG, 22331 Hamburg, Germany) under the following conditions: initial denaturation at 95.0 C for 5 minutes, followed by 35 cycles of 94.0 C for 30 seconds, 63.5 C for 30 seconds, and 72.0 C for 40 seconds. PCR products were sequenced on an ABI 3730XL DNA Analyzer (Applied Biosystems, Foster City, CA, USA). The reference gDNA sequence was obtained from the UCSC Genome Browser (http://genome.ucsc.edu/). All sequences were compared to the referenced gDNA sequence, using Chromas software (Technelysium Pty Ltd, Brisbane, Australia).

**Table 1:** Clinical presentations of SCA3/MJD patients.

Clinical features	Genotypes		
	G/G	G/A	A/A
Age at onset Mean ± SD (range)^a^			
	37.70 ± 9.90 (17–59)	36.19 ± 9.84 (14–57)	34.09 ± 9.45 (15–53)
Duration Mean ± SD (range)			
	10.16 ± 3.87 (5–25)	11.77 ± 5.14 (5–25)	11.95 ± 4.30 (7–20)
Ataxia symptoms, No (%)	55 (100%)	83 (100%)	32 (100%)
Pyramidal sign	15 (8.82%)	22 (12.94%)	14 (8.23%)
Extrapyramidal signs	10 (5.88%)	17 (10.00%)	5 (2.94%)

a. Before adjusting for the mean size of expanded CAG repeats in the patients

### Statistics

Given their complete correlation, we tested only one of the two SNPs for association, rs709930. Frequencies of the rs709930 genotypes and alleles in SCA3/MJD patients and healthy controls were calculated, and analyzed for statistically significant differences using the Chi-square test. The difference of the distribution of CAG repeat lengths between the presence and the absence of the rs709930 A allele was analyzed using the Mann-Whitney *U* test. The risk of developing SCA3/MJD before age 36 years (mean AO for the present series: 36.29±9.81) among patients with the rs709930 A allele was estimated as an odds ratio by logistic regression analysis, with AO≤36 years versus AO＞36 years as the dependent variable. We used multivariate linear regression analysis to estimate the effect of several potential variables on AO, including the (CAG)n tract sizes, the genotypes of the rs709930, and gender. AO for the rs709930 genotypes was adjusted for the mean CAG repeat lengths in the expanded *ATXN3* allele after fitting a linear regression model. All analyses were performed using the SPSS Statistics software (version 17.0). A *P*-value ≤0.05 was considered statistically significant.

## Results

Three rs709930 genotypes (G/G, G/A, A/A) appeared both in SCA3/MJD patients and healthy controls. rs709930 genotypes and alleles frequencies were statistically significantly different between patients and controls (both *p* = 0.001,*α* = 0.05) (Table 2).

**Table 2:** Genotype and allele frequencies of rs709930 in SCA3/MJD patients and healthy controls.

Group	Genotype, NO. (%)				Allele (%)	
	Total	G/G	G/A	A/A	G	A
Patients	170	55(32.4)	83(48.8)	32(18.8)	56.8	43.2
Gender						
Female	73	25(34.25)	33(45.20)	15(20.55)	56.85	43.15
Male	97	30(30.93)	50(51.55)	17(17.53)	56.70	43.30
AO≤36 years old	89	22(24.72)	46(51.68)	21(23.60)	50.56	49.44
AO＞36 years old	81	33(40.74)	37(45.68)	11(13.58)	63.58	36.42
Controls	200	90(45.0)	95(47.5)	15(7.5)	68.8	31.3
Gender						
Female	93	45(48.39)	42(45.16)	6(6.45)	70.97	29.03
Male	107	45(42.06)	53(49.53)	9(8.41)	66.82	33.18

**Fig 1:**
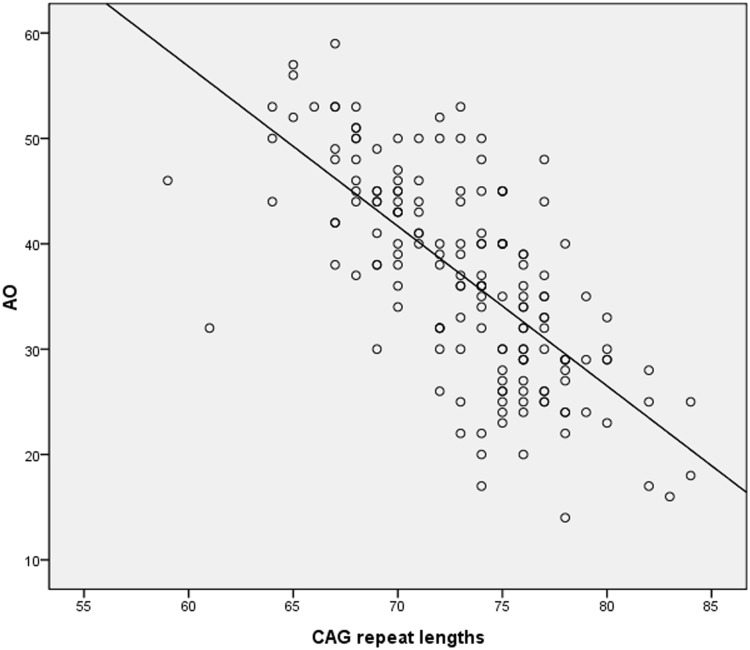
The association of expanded CAG repeats in the expanded *ATXN3* gene with age at onset (AO) in SCA3/MJD patients. The X-axis indicates the expanded CAG repeat lengths and the Y-axis denotes AO in years. AO of SCA3/MJD is inversely correlated with the length of CAG repeat (*r* = －0.694, *p* = 0.000).

AO of SCA3/MJD was negatively correlated with the CAG repeat lengths in the expanded *ATXN3* gene, confirming previous findings [1] (present series, *r* = －0.694, *p* = 0.000) (Fig. 1). However, no significant difference was detected in the distribution of the CAG repeat lengths between the presence and the absence of the rs709930 A allele (*p* = 0.628). A statistically significant difference was found between the AO≤36 years group and the AO＞36 years group, demonstrating the association between the presence of the A allele and an earlier AO (*p* = 0.001, OR = 2.660, 95% CI = [1.506, 4.700]) that is not mediated by a change in the number of CAG repeats.

Given the relatively earlier onset observed for carriers of the rs709930 A allele, the role of presence or absence of the rs709930 A allele was analyzed. When rs709930 A allele status was taken into consideration, the percentage of onset variance explained increased from 44.6% to 46.5% (F = 74.482, *p* = 0.000). In this series of patients, after adjusting for the size of expanded CAG repeats, the presence of rs709930 A allele decreased the AO by approximately 2–4 years (Table 3). When gender was considered as a variable, the model was not significantly improved.

**Table 3:** Age at onset in SCA3/MJD patients with rs709930.

Characteristics	Genotypes		
	G/G (n = 55)	G/A (n = 83)	A/A (n = 32)
Age at onset (y)			
Mean ± SD (range)	37.70 ± 9.90 (17–59)	36.19 ± 9.84 (14–57)	34.09 ± 9.45 (15–53)
Adjusted, mean (SE)^a^	38.17 (0.98)	35.94 (0.79)	34.06 (1.28)
Number of CAG repeat lengths			
Expanded, mean ± SD (range)	73.40 ± 3.68 (67–82)	72.94 ± 4.74 (59–84)	73.09 ± 4.74 (64–84)

a. Adjusted for the mean size of expanded CAG repeats in the patients; SD, standard deviation; SE, standard error.

## Discussion

In this study, we found statistically significant differences between the frequencies of rs709930 alleles and genotypes SCA3/MJD patients and healthy controls. We also identified an association between AO of SCA3/MJD and rs709930 (and rs910369) genotype. When the status of rs709930 A allele (and rs910369 T allele) was taken into account, the percentage of total variance explained increased by nearly 2%. However, in our study, the expanded CAG repeats that still came to less than 50% of AO variance, which is lower than previously reported [7, 8]; differences in ancestry and sample size may be responsible for the discrepancy. In addition to the CAG repeat lengths in expanded alleles, AO of SCA3/MJD was significantly earlier in patients with the rs709930 A/A and G/A genotypes (rs910369 T/T and G/T genotype) than those with the rs709930 G/G genotype (rs910369 G/G genotype). Moreover, the presence of rs709930 A allele (and rs910369 T allele) decreased the AO by nearly 2–4 years, which showed that variance of AO might be affected by SNPs of disease-causing genes or disease-associated genes as modifiers.

In addition to variation in the number of CAG repeats in causative genes, the variability of AO in PolyQ diseases can also be explained by the effects of modifier sister genes: *ATXN2*, *ATN1* and *HTT* in SCA3/MJD; *ATXN1* and *ATXN3* in SCA6; and *ATXN3* and *TBP* in SCA7 [7], as well as other modifiers like *APOE* in SCA3/MJD [11, 12]. Additionally, investigation on variation in the 5’ UTR region of *ATXN3* was performed to evaluate the potential to influence SCA3/MJD phenotype, however, no improvement on the explanation of AO variance was observed [13]. Here, we firstly found that two novel SNPs in 3’UTR of *ATXN3*, so called “modifying SNPs”, might have effect on AO of SCA3/MJD, which may provide new insights to explore other modifying factors in PolyQ diseases. Due to a relatively small sample size, these findings should be validated based on a larger dataset, together with functional assays to elucidate the underlying mechanisms. The upcoming investigation on other non-coding region of *ATXN3* and other causative genes in PolyQ diseases to explore more “modifying SNPs” may be helpful to expand the spectrum of modifiers in PolyQ diseases, which would be beneficial to better understanding of the relationship among causative genes, genetic modifiers, and phenotypes.
